# Knowledge of Pregnant Women on Mother-to-Child Transmission of HIV in Meket District, Northeast Ethiopia

**DOI:** 10.1155/2015/960830

**Published:** 2015-02-09

**Authors:** Tesfaye Birhane, Gizachew Assefa Tessema, Kefyalew Addis Alene, Abel Fekadu Dadi

**Affiliations:** ^1^Meket District Health Office, Meket, Ethiopia; ^2^Department of Reproductive Health, Institute of Public Health, University of Gondar, P.O. Box 196, Gondar, Ethiopia; ^3^Department of Epidemiology and Biostatistics, Institute of Public Health, University of Gondar, P.O. Box 196, Gondar, Ethiopia

## Abstract

Knowledge of pregnant women on the three periods of mother-to-child transmission (MTCT) of HIV has implication for child HIV acquisition. This study aims to assess the knowledge of pregnant women on mother-to-child transmission of HIV and to identify associated factors in Meket district, northeast Ethiopia. Logistic regression models were fitted to identify associated factors. Adjusted odds ratios (AOR) with 95% confidence intervals (CI) were used to determine the presence and strength of association. About one-fifth (19%) of women were knowledgeable on mother-to-child transmission of HIV (95% CI: 15.5%, 22.4%). Being urban resident (AOR: 2.69, 95% CI: 1.48, 4.87), having primary education (AOR: 2.41, 95% CI: 1.03, 5.60), reporting receiving information on HIV from health care providers (AOR: 3.24, 95% CI: 1.53, 6.83), having discussion with partner about mother-to-child transmission of HIV (AOR: 2.64, 95% CI: 1.59, 4.39), and attending antenatal care (AOR: 5.80, 95% CI: 2.63, 12.77) were positively associated with increased maternal knowledge of mother-to-child transmission of HIV. Knowledge of mother-to-child transmission of HIV among pregnant women was low. Providing information, especially for rural women and their partners, is highly recommended.

## 1. Background

Vertical transmission of Human Immunodeficiency Virus (HIV) is still a major challenge in the world, especially in developing countries [1]. A report in 2012 reported about 35.3 million people are living with HIV of which 2.3 million are new infections whereas an estimated 3.3 million infected people are less than 15 years of age. Worldwide, there are about 6,300 new infections and 700 HIV-related deaths daily in 2012. Sub-Saharan Africa remains the region most heavily affected by HIV [2].

Without any intervention, the risk of a baby getting HIV infection from an infected mother ranges from 15% to 25% in the developed nations and from 25% to 35% in developing countries. HIV transmission rate and timing are estimated to be 5% to 10% during pregnancy, 10% to 15% during delivery and 5% to 20% through breast-feeding. In general mother to child transmission contributes 15-45% of HIV acquisition for children [3].

The national adult HIV prevalence in Ethiopia is 1.2% [4]. The national accelerated emergency plan includes three targeted objectives, that is, reaching 90% of pregnant women with access to antenatal care services, ensuring that all pregnant women have access to delivery by a skilled attendant, and providing antiretroviral prophylaxis to at least 80% of HIV-positive pregnant women [5].

It is estimated that 138, 906 children less than 15 years are living with HIV in 2014. There are an estimated 3,886 new infections each year due to mother-to-child transmission [4]. However, timely interventions can reduce mother-to-child transmission to 2–5% [3, 6, 7]. A global target has also been established to be achieved by the year 2015, that is, elimination of new HIV infections among children and prolonging the lives of the mothers with HIV [1].

According to Ethiopian Demographic and Health Survey (EDHS) report, about three-quarters of reproductive aged women know that HIV can be transmitted to a baby through breastfeeding [8].

The prevention of Mother-To—Child-Transmission (MTCT) of HIV is dependent on the knowledge of the mothers of the timing of possible transmission periods. However, knowledge of women on transmission periods of HIV from mother to child varies from country to country and has not been measured in Ethiopia at community level.

Different studies reported that sociodemographic factors like age [9], urban living [10], higher educational level [11], and being house wife [12] as factors that affect mothers' knowledge of MTCT of HIV. Studies conducted in southern and northwest Ethiopia [10, 12, 13] reported that gravidity, parity, antenatal care (ANC) visits, and male partner discussion are factors associated with good knowledge of mothers on MTCT of HIV.

Maternal knowledge on MTCT is a corner stone of effective implementation of the World Health Organization (WHO) recommendation of the four-pronged approach to reduce mother-to-child transmission of HIV [1].

Despite the large challenge of vertical transmission of HIV, there were also limited community-based studies on women knowledge on mother-to-child transmission of HIV. Hence, this study attempts to fill the gap through assessing the level of knowledge of MTCT of HIV and its associated factors at Meket district, Northeast Ethiopia.

## 2. Methods

### 2.1. Study Design, Population, and Setting

A community-based cross-sectional study design was conducted in Meket district, northeast Ethiopia, from March 8 to 21, 2014. Meket district is located 665 km north of Addis Ababa, the Ethiopian capital city. The district has an estimated population size of 254,520 of which 59,939 are reproductive aged women, and an estimated 8,246 were pregnant women. Those pregnant women are living in Meket district were constituted our study population.

### 2.2. Sample Size and Sampling Procedure

Sample size was determined using single population proportion formula with the assumptions of 95% level of confidence, 12% proportion of knowledgeable women on MTCT of HIV [12], 4% of margin of error, and design effect of two. Finally, considering a non-response rate of 10%, the total sample size was calculated to be 556. Multistage stratified sampling technique was used to select the study participants. In the district, there are two urban and 46 rural kebeles. Hence, in the first step, eight rural kebeles were randomly selected; however, since they are few, all the urban kebeles were included. On the second stage, 79 pregnant women from urban kebeles and 477 pregnant women from rural kebeles were randomly selected.

### 2.3. Operational Definitions

In the present study, pregnant woman was regarded as being knowledgeable on MTCT if she correctly identified the three different modes/periods of MTCT of HIV; otherwise she was classified as nonknowledgeable. Comprehensive knowledge of HIV was also measured if a pregnant woman correctly identified three modes of transmission of HIV (unsafe sexual practice, blood transfusion, and MTCT) and recognized two common misconceptions.

Comprehensive knowledge about HIV/AIDS was measured after posing the following questions: (1) knowing that condom use and limiting sex partners to one uninfected partner are HIV prevention methods, (2) being aware that a healthy-looking person can have HIV, and (3) rejecting the two most common local misconceptions, that is, HIV/AIDS can be transmitted through mosquito bites and by supernatural means in Ethiopia [8].

### 2.4. Data Collection Procedures

Data were collected using pretested, structured, and interviewer administered questionnaire. The questionnaire was prepared after reviewing relevant literatures. Five female nurses supervised by two BSc health professionals collected the data. For eligible women who were not at home during our first attempt, the interviewers revisited the participant's home at least two times before excluding the participant.

Training was given to the data collectors about informed consent, techniques of interviewing, data collection procedures, and different sections of the questionnaire. Supervisors and principal investigators checked the questionnaire on its completeness and consistency on the daily basis.

### 2.5. Data Processing and Analysis

The data were entered into EPI info version 3.5.3 statistical software and then sorted, cleaned, and analyzed by using SPSS version 20 statistical package. Descriptive statistics were done to describe the study participants in relation to relevant variables. Both bivariate and multiple logistic regression analyses were carried out to see the effect of sociodemographic factors, maternal condition factors, and other factors on the knowledge of MTCT of HIV and to control cofounding. Odds ratios with 95% CI were computed to identify factors associated with mothers' MTCT knowledge.

### 2.6. Ethical Consideration

Ethical clearance was obtained from the Research and Ethical Review Committee (REC) at the Institute of Public Health, College of Medicine and Health Science of University of Gondar. Permission letter was secured from Meket District Health Office. Written informed consent was taken from each study participant after reading the consent form. The purpose and benefit of the study and their right to withdraw at any time were also delivered to each participant prior to the interview. Confidentiality of the information was maintained throughout by using anonymity identifiers, keeping their privacy by interviewing them individually.

## 3. Results

### 3.1. Sociodemographic Characteristics of Pregnant Women

Five hundred forty-two pregnant women participated in the study (97.5% response rate). The majority (85.4%) were rural dwellers. The mean age of the study participants was 29.45 years (SD = 5.4). Four hundred and sixty (84.9%) were married, 196 (36.2%) were able to read and write, and nearly four-fifths (80.1%) were homemaker (Table 1).

### 3.2. Reproductive Health Related Characteristics and MTCT of HIV Knowledge

One hundred sixty-one (19.7%) were pregnant for the first time. More than half (57.6%) had ANC during their current pregnancy. Nearly two-thirds (63.8%) had received information about HIV/AIDS from health care providers.

Half (51.8%) of the respondents received information about HIV, antenatal care (65.7%), mother-to-child transmission of HIV (40.6%), and infant feeding with their partners (21.4%) (Table 2).

### 3.3. Knowledge of Pregnant Women on MTCT

One hundred three (19%) (95% CI: 15.5%, 22.4%) were knowledgeable on MTCT of HIV. Most (84.5%) heard about mother to child transmission of HIV. Among those who heard MTCT, more than two-thirds (70.7%) mentioned labor/delivery as a time of HIV transition from mother to child. 225 (41.5%) pregnant women identified at least two periods of mother-to-child transmission of HIV. Nearly two-thirds (63.8%) had comprehensive knowledge on HIV/AIDS, and another equivalent proportion of women heard about PITC (Table 2).

### 3.4. Factors Associated with Knowledge of Pregnant Women on MTCT of HIV

In multivariable analysis, higher levels of maternal education status, having received information about HIV from health professionals, and reported discussion of MTCT and ANC with their partners were positively associated with knowledge of mother-to-child transmission of HIV. Those women who live in the urban settings were about three more like to be knowledgeable than their rural counterparts (AOR: 2.69, CI (1.48, 4.87)). Those literate mothers were about three times more likely to be knowledgeable than who did not read and write (AOR: 3.25, CI (1.55, 6.78)). Likewise, a woman was 2.41 times more likely to be knowledgeable if she had completed primary school as compared to those who did not read and write (AOR: 2.41, CI (1.04, 5.60)).

Pregnant women who received information on HIV from health care providers were about three times more likely to be knowledgeable than women who had not received information (AOR: 3.24, CI (1.54, 6.83)). Women who had discussions with their partner were more likely to be knowledgeable than those who had not (AOR: 5.80, CI (2.63, 12.78)). Correspondingly, mothers who discussed MTCT with their partners were more likely to be knowledgeable than those who had not (AOR: 2.64, CI (1.59, 4.39)) (Table 3).

## 4. Discussion

Being knowledgeable on MTCT of HIV and the fact that the risk of transmission can be reduced by using antiretroviral drugs are critical in reducing MTCT of HIV. This can contribute greatly towards the achievement of the Millennium Development Goals related to HIV.

This study revealed that 19% (95% CI: 15.5%, 22.4%) of respondents were knowledgeable on MTCT of HIV. This result is in line with a cross-sectional study conducted at Temeke District Hospital, Dar Es Salaam (15.7%) [14]. However, it is higher than that of studies done in southern Ethiopia (11.5%) and Gondar town (8.5%) [10, 13] but lower than a health institution based study in Debre Markos town, Ethiopia (42.3%) [15]. This could be due to the difference in the study setting and accessibility of health facilities.

In the present study, nearly two-thirds of pregnant women had comprehensive knowledge on HIV/AIDS which is higher than studies in Yaoundé (23%) [16], the Ethiopian Demographic and Health Survey (19%) [8], and a study in Gondar town (59.8%) [10].

Knowledge of pregnant women on MTCT of HIV among pregnant women was significantly varied based on their place of residence. Those pregnant women residing in urban areas were more likely to be knowledgeable when compared to the rural residents. This finding is in line with studies conducted at Gondar and Hawassa towns in Ethiopia [10, 13]. It might be due to the rural location and geographical inaccessibility and poor availability of nearby health services, compared with urban areas. This could also be partly explained due to the presence of media exposure amongst urbanites.

Educated pregnant women who were able to read and write were more likely to be knowledgeable than those who were unable to read and write. This supports the government attempt to address adult informal education. Pregnant women with primary education were also more likely to be knowledgeable than those who were unable to read and write. This result is in line with a previous study conducted in southern Ethiopia [12]. This could be because when the women become educated their access to information is also increased. With this regard, they might have access to print media exposure.

In this study, pregnant women who discussed and received information about HIV/AIDS from health care providers were more knowledgeable. They were found to be three times more likely to be knowledgeable than those who had not.

Spouse discussion on antenatal care follow-up was also positively associated with knowledge of MTCT. Those pregnant women who had discussions with their partners were six times more likely to be knowledgeable than those who had not discussed the issue. This is similar to reports from other studies [12, 17]. This might be explained due to male partners possessing better knowledge on HIV transmission and eventually transfer this information to these pregnant women if discussion is triggered.

Pregnant women may receive information from a variety of sources about health services. Spouses having delivered information and participated in discussions about MTCT of HIV with their wives (40.6%) were associated with good knowledge of the subject. Accordingly, pregnant women who had discussion with their partners were more than two times more likely to have good knowledge of MTCT. This might be because partner discussion in this regard could enhance their knowledge.

This study tried to assess pregnant women who did not attend health care facilities for ANC and HIV concerning their knowledge about MTCT of HIV. However, because of financial and time constraints, this study did not include the knowledge part of prevention of mother-to-child transmission of HIV.

## 5. Conclusions

Despite many efforts, the knowledge of pregnant women on mother-to-child transmission of HIV is low. If pregnant woman resides in urban environment, she attends school, if she receives information on HIV from health care providers, and if she attends antenatal care, she is more likely to be knowledgeable on MTCT of HIV. Strengthening women education and by reaching previously inaccessible parts of the community, integration of HIV, prevention of MTCT, and ANC service, is highly recommended. Moreover, strengthening discussion of MTCT with spouses is important.

## Figures and Tables

**Table 1:** Selected sociodemographic characteristics of respondents, Meket district, northeast Ethiopia, 2014 (*n* = 542).

Variables	Frequency	Percent
Age (years)		
15–24	99	18.3
25–34	326	60.1
35–49	117	21.6
Residence		
Urban	79	14.6
Rural	463	85.4
Marital status		
Married	460	84.9
Single	27	5
Divorced	55	10.1
Educational status		
Unable to read and write	176	32.5
Able to read and write only	196	36.2
Primary	127	23.4
Secondary and above	43	7.9
Occupation		
Housewife	434	80.1
Student	26	4.8
Merchant	55	10.1
Government employee	27	5.0
Income (ETB)		
≤450	458	84.5
451–999	77	14.2
≥1000	7	1.3

**Table 2:** Reproductive health related characteristics and information received from health care providers, Meket district, northeast Ethiopia, 2014 (*n* = 542).

Variables	Frequency	Percent
Number of pregnancies		
1	161	29.7
2-3	276	50.9
4+	105	19.4
Gestational age		
≤16	10	1.9
17–24	134	24.7
25–35	333	61.4
≥36	65	12.0
Antenatal visit		
Yes	312	57.6
No	230	42.4
Number of ANC visit (*n* = 312)		
1	122	39.1
2-3	176	56.4
4+	14	4.5
Received information from health care providers		
On HIV		
Yes	346	63.8
No	196	36.2
On antenatal care		
Yes	304	56.1
No	238	43.9
On MTCT		
Yes	284	52.4
No	258	47.6
On infant feeding		
Yes	181	33.4
No	361	66.6
Comprehensive knowledge of HIV/AIDS		
Yes	346	63.8
No	196	36.2
Heard of PITC		
Yes	345	63.7
No	197	36.3
Heard about MTCT		
Yes	458	84.5
No	84	15.5
Know the means of transmission on MTCT (*n* = 458)		
During pregnancy	309	67.5
During labor/delivery	324	70.7
During breast feeding	251	54.8
Exact timing of MTCT answered by women		
None	84	15.5
One	130	24.0
Two	225	41.5
Three	103	19.0

**Table 3:** Crude and adjusted odds ratios (OR) and 95% confidence intervals (CI) of factors associated with knowledge of mothers on MTCT of HIV among pregnant women, Meket district, 2014 (*n* = 542).

Variables	Knowledge on MTCT		COR (95% CI)	AOR (95% CI)
	Yes	No		
Residence				
Urban	31	48	3.51 (2.01, 5.88)	2.69 (1.48, 4.87)^**^
Rural	72	391	1.0	1.0
Age				
15–24	34	65	1.0	
25–34	56	270	0.40 (0.23, 0.65)	
35–49	13	104	0.24 (0.11, 0.48)	
Education				
Unable to read and write	13	163	1.0	1.0
Able to read and write	49	147	4.18 (2.18, 8.01)	3.25 (1.55, 6.79)^**^
Primary	31	96	4.05 (2.02, 8.11)	2.41 (1.03, 5.60)^**^
Secondary and above	10	33	3.80 (1.53, 9.39)	2.05 (0.71, 5.88)
ANC information from HP				
Yes	84	220	4.40 (2.58, 7.49)	
No	19	219	1.0	
HIV information from HP				
Yes	92	254	6.09 (3.16, 11.70)	3.24 (1.53, 6.83)^**^
No	11	185	1.0	1.0
MTCT discussion with husband				
Yes	67	153	3.48 (2.21, 5.45)	2.64 (1.59, 4.39)^**^
No	36	286	1.0	1.0
ANC discussion with husband				
Yes	95	261	8.10 (3.84, 17.08)	5.80 (2.63, 12.77)^**^
No	8	178	1.0	1.0

^**^Statistically significant at *P* value <0.05.
